# Rapid Improvement in Neck Disability, Mobility, and Sleep Quality with Chronic Neck Pain Treated by Fu's Subcutaneous Needling: A Randomized Control Study

**DOI:** 10.1155/2022/7592873

**Published:** 2022-09-30

**Authors:** Ching-Hsuan Huang, Lung-Hung Tsai, Mao-Feng Sun, Zhonghua Fu, Jian Sun, Li-Wei Chou

**Affiliations:** ^1^Department of Chinese Traumatology Medicine, China Medical University Hospital, Taichung 404332, Taiwan; ^2^Graduate Institute of Chinese Medicine, China Medical University, Taichung 404332, Taiwan; ^3^Drug Development Center, China Medical University, Taichung 404332, Taiwan; ^4^Research Center for Cancer Biology, China Medical University, Taichung 404332, Taiwan; ^5^Graduate Institute of Acupuncture Science, China Medical University, Taichung 404332, Taiwan; ^6^Division of Chinese Acupuncture Medicine, China Medical University, Taichung 404332, Taiwan; ^7^Institute of Fu's Subcutaneous Needling, Beijing University of Chinese Medicine, Beijing 100029, China; ^8^Clinical Medical College of Acupuncture & Moxibustion and Rehabilitation, Guangzhou University of Chinese Medicine, Guangzhou 510405, China; ^9^Second Clinical Medical College, Guangzhou University of Chinese Medicine, Guangzhou 510006, China; ^10^Department of Physical Medicine and Rehabilitation, China Medical University Hospital, Taichung 404332, Taiwan; ^11^Department of Physical Therapy and Graduate Institute of Rehabilitation Science, China Medical University, Taichung 406040, Taiwan; ^12^Department of Physical Medicine and Rehabilitation, Asia University Hospital, Asia University, Taichung 413505, Taiwan

## Abstract

**Background:**

Chronic neck pain is a common musculoskeletal disorder caused by overuse of neck and upper back muscles or poor posture, and it is commonly combined with a limited range of motion in the neck and shoulders. Most cases will recover within a few days; however, the symptoms often recur easily. Fu's subcutaneous needling (FSN) is a new therapeutic approach used to treat patients with chronic neck pain. However, there is no solid evidence to support the effectiveness of FSN on chronic neck pain and disability.

**Methods:**

Participants (*n* = 60) with chronic neck pain for more than 2 months with pain intensity scored by visual analog scale (VAS) more than five were enrolled in this trial. Participants were equally randomized into the FSN or transcutaneous electrical nerve stimulation (TENS) group who received interventions once a day on day 1, day 2, and day 4. They were assessed by outcome measurements during pre- and post-treatment and followed up for 15 days.

**Results:**

The VAS was immediately reduced in the FSN and TENS groups and sustained for 15 days of follow-up (all *P* < 0.001). The immediate effects were also observed as the pressure pain threshold increased in the FSN group on day 2 (*P*=0.006) and day 4 (*P*=0.023) after treatment, and tissue hardness decreased by FSN on day 1 and day 2 after treatment (both *P* < 0.001). FSN and TENS treatment improved neck disability and mobility; moreover, FSN promoted participants to receive better sleep quality, as determined by PSQI assessment (*P*=0.030). TENS had no benefit on sleep quality.

**Conclusion:**

FSN was able to relieve pain and relax muscle tightness. Notably, FSN significantly improved neck disability and mobility and enhanced sleep quality. These findings demonstrated that FSN could be an effective alternative treatment option for patients with chronic neck pain. Clinical Trial Registration: ClinicalTrials.gov Identifier: NCT03605576, registered on July 30, 2018.

## 1. Introduction

The number of people with neck pain increases daily, and it has become a serious social problem in modern life. Acute neck pain is caused by overuse of the neck and upper back muscles or poor posture or injury, and it is usually combined with a limited range of motion [1]. Symptoms are often resolved without any treatment in a few days. However, most people experience recurrence of symptoms; pain that persists for more than 2 months without improvement is categorized as chronic neck pain that leads not only to upper back pain but also to functional decline that affects daily life, work, and sleep quality [1]. In 2017, more than 280 million cases of neck pain were reported, and the trend of age-standardized point prevalence did not decrease from 1990 [2]. Furthermore, about 10% of the population live with neck disability due to chronic neck pain [1–3]. The prevalence is greater in females than in males [4]. The highest prevalence, annual incidences, and years lived with disability from neck pain have been reported in developed regions (such as east Asia, western Europe, North Africa, and the Middle East) and high-income areas (e.g., North America) [2]. These epidemiological studies showed the urgent need to investigate new strategies for chronic neck pain treatment.

Treatments for chronic neck pain aim to relieve pain and recover functional disability. In general, rest, good posture, and intake of non-steroidal anti-inflammatory drugs are good options for managing neck pain [5]. However, some patients experience recurrent symptoms that persist without improvement. To alleviate these symptoms, some clinical practices are routinely used for chronic neck pain treatment. Transcutaneous electrical nerve stimulation (TENS) is a non-invasive therapy that is widely used in clinics. TENS treatment over the acupuncture points plus infrared irradiation can effectively reduce neck pain [6]. Furthermore, needle therapy is an effective alternative treatment for chronic neck pain. For example, remote acupuncture on TE 5 (*Waiguan*) and LI 11 (*Quchi*) has been reported as an effective treatment to manage chronic neck pain caused by myofascial trigger points (MTrPs) on the upper trapezius muscle [7]. Deep dry needling on active MTrPs provides a beneficial effect on pain relief and neck disability on chronic neck pain [8]. Fu's subcutaneous needling (FSN) is an advanced acupuncture that is applied for the treatment of MTrP-induced musculoskeletal disorders [9]. FSN is manipulated by using a disposable needle penetrating the skin of the non-diseased area and targeting the subcutaneous layer rather than the dermis or muscle layer. The swaying and reperfusion approach are the effective features of FSN, distinct from traditional acupuncture or dry needling [9, 10]. These features support FSN as an acceptable and popular needle therapy. Recently, FSN was demonstrated as an effective therapy for lateral epicondylalgia treatment without adverse effects [11]. However, scientific-based evidence to support the effects of remote FSN on chronic neck pain is currently lacking.

In the present study, we evaluated the effectiveness of FSN on chronic neck pain by measuring visual analog scale (VAS), pressure pain threshold (PPT), tissue hardness (TH) meter, neck range of motion (NROM), neck disability index (NDI), and Pittsburgh sleep quality index (PSQI) as outcome measurements. Three treatment sessions were performed on day 1, day 2, and day 4, with assessments before each treatment session and immediately after treatment, as well as on day 8 and day 15 for follow-up.

## 2. Materials and Methods

### 2.1. Participants

Subjects who participated in this study were enrolled from the Departments of Physical Medicine and Rehabilitation and Acupuncture in the China Medical University Hospital according to an open-label, randomized controlled trial. This study was approved by the Institutional Review Board of the China Medical University Hospital (CMUH107-REC2-031) and registered with ClinicalTrials.gov (Identifier: NCT03605576). All patients had completed their informed consent to participate in this study, and the research was conducted in accordance with the principles of the Declaration of Helsinki.

The inclusion criteria were based on (1) adults older than 20 years old; (2) having chronic neck pain for more than 2 months, as defined by the International Association of the Study of Pain, updated in 2011 [12], and VAS greater than 5 points; (3) patients with myofascial pain on the upper back; and (4) pain that was not effective for previous medication or physical therapy. Participants were excluded based on the criteria of (1) contraindications for FSN or TENS treatment, such as serious medical problems, recent trauma, or pregnancy; (2) history of drug abuse (including excess alcohol) that affected pain assessments; (3) received neck, upper back, or upper and lower limb surgery; (4) people with central or peripheral nerve disease; (5) cognitive dysfunction could not be matched with the experimenter; and (6) people with cardiac pacemakers and epilepsy, because electrode patches could not be placed on the skin.

**Figure 1:**
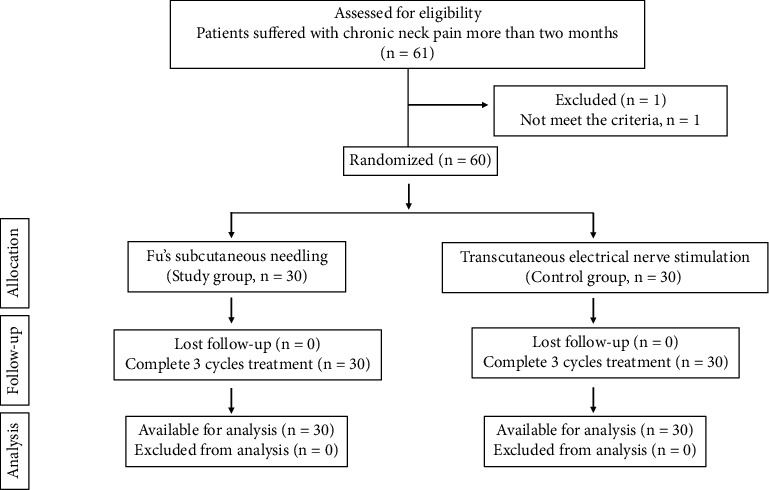
Consort flow diagram.

**Figure 2:**
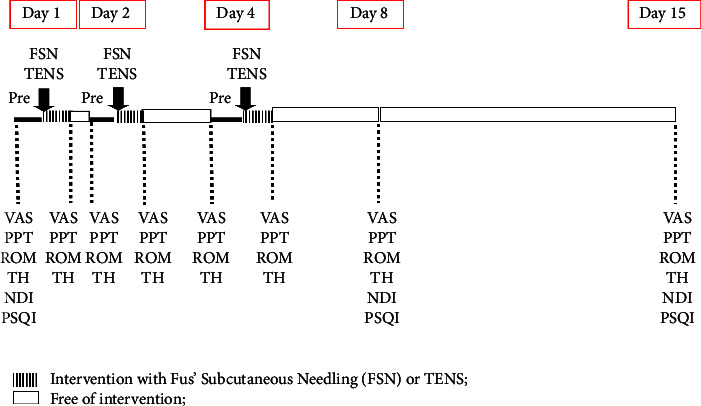
Study design. All participants were treated with FSN or TENS as described in Materials and Methods. Pre: before intervention; FSN: Fu's subcutaneous needling; TENS: transcutaneous electrical nerve stimulation; VAS: visual analog scales; PPT: pressure pain threshold; ROM: active neck range of motion; TH: tissue hardness; NDI: neck disability index; PSQI: Pittsburgh sleep quality index.

Participants were randomly divided into FSN as the experimental group or TENS as the control group by a raffle system and allocated to the FSN or TENS group (Figure 1). A total of 61 participants were enrolled, but one participant was excluded because of VAS smaller than 5. The participants (60 patients) were divided and allocated into two arms: an experimental group who underwent FSN treatment (30 patients) and a placebo group who underwent TENS treatment (30 patients) via raffle (Figure 1). Every participant received the intervention of FSN or TENS. Three treatment sessions in this experiment were performed on day 1, day 2, and day 4, with assessments before each treatment session and immediately after treatment, as well as the following day 8 and day 15 for follow-up (Figure 2). All the treatments were conducted by the same acupuncturist who worked in the medical center in Taiwan for more than 5 years.

### 2.2. Intervention Procedures

**Figure 3:**
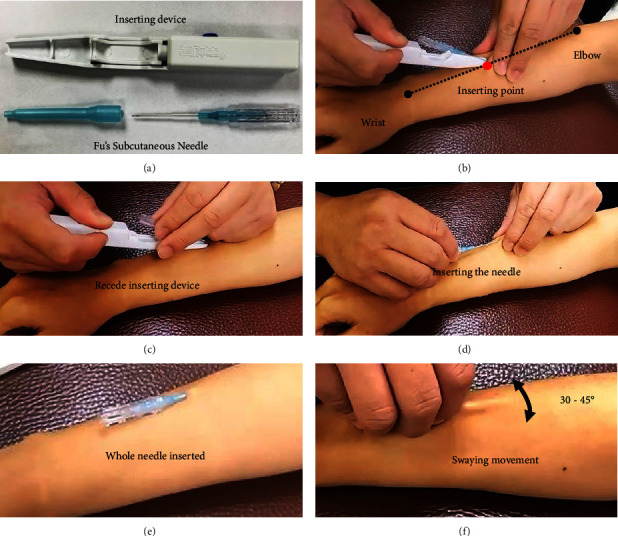
Procedure of Fu's subcutaneous needling. FSN was performed with a disposable Fu's subcutaneous needle (bottom) and inserting device (top) (a). The inserting point was on the midpoint between wrist and the elbow of the affected forearm (b). Holding the Fu's subcutaneous needle and receding inserting device (c). Inserting the needle into subcutaneous layer (d) until the whole needle is inserted (e). Swaying the needle in a 30-to-45-degree movement (f).

**Figure 4:**
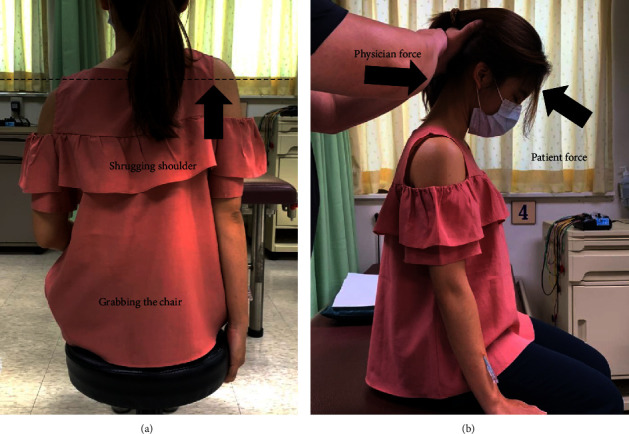
Reperfusion approach of Fu's subcutaneous needling. The participant was asked to grab the chair and shrug her shoulder on the same affected arm (a) and extend the neck with resistance by the acupuncturist's push (b) for the contraction of the upper trapezius muscle. The horizontal dashed line indicated the right shoulder shrugging.

Participants in the experimental group were treated by using a disposable Fu's subcutaneous needle (Nanjing FSN Medical Co., Ltd., Jiangsu, China) on the radial aspect of the forearm extensors muscle. The needle was inserted into the subcutaneous layer with the whole needle body by holding the inserting device (Figure 3(a)). The insertion point was on the midpoint of the extensor muscle of the affected forearm (the center between the midpoint of cubital crease to elbow tip to the center of transverse crease of the wrist, Figure 3(b)). To ensure that the needle was inserted into the subcutaneous layer rather than the dermis or muscle layers, participants were asked for no pain sensations or soreness during the whole process of insertion. The core needle receded, and the protuberance of the soft tube seat was fixed in the slot of the core seat so that the needle tip was no longer exposed outside, followed by starting a swaying movement (Figures 3(c)–3(e)). The tip of the needle should be maintained at the same horizontal level during swaying by using the thumb and the middle finger to hold the core base, and the index finger and the ring finger were separated on the left and right side of the middle finger to sway in a seesaw-like sector one after the other (Figure 3(f)). Time and frequency of swaying was 50 times within 30 s. After swaying, the participants were asked to shrug their shoulders and raise their head for 10 s and then rest for the same intermission (Figure 4(a)). In this step, the physician could help participants perform the exercise of contraction of the upper trapezius muscle with resistance (Figure 4(b)). The cycle was repeated up to three times for 2 min. After finishing the two actions called the “reperfusion approach,” with FSN embedded subcutaneously, we removed the needle afterward.

Participants in the TENS group were treated with transcutaneous electrical nerve stimulator (Well-Life Healthcare Limited, Taiwan), with the electrodes attached to acupoints TE 5 (*Waiguan*) and LI 11 (*Quchi*), according to the guidance of WHO. The treatment parameters were set to pulse width of 200 *μ*s, frequency of 200 Hz, and continuous wave for 20 min.

### 2.3. Outcome Measurements

#### 2.3.1. Visual Analog Scales

VAS is a subjective tool that is commonly used to evaluate pain intensity [13, 14]. Participants were subjected to evaluations of the score of pain severity from no pain (score 0) to intolerable pain (score 10) in a 10-cm-long scale. The results were recorded in every pre-treatment (pre-Tx) and post-treatment (post-Tx) on day 1, day 2, and day 4 and followed up to day 8 and day 15 (Figure 2).

#### 2.3.2. PPT for MTrPs of Upper Trapezius Muscles

We used a semi-objective tool by Pressure Algometry (OE-220, ITO CO., Ltd., Tokyo, Japan) as Fischer's methods to evaluate PPT [15, 16]. First, the physician found the MTrP of upper trapezius muscles and marked the point. The metal probe of pressure algometry was attached vertically to the MTrP, and the press was increased by 1 kg/s. When the participant felt uncomfortable or in pain gradually, this point indicated the threshold of latent MTrP. A point of intolerable pain indicated the threshold of active MTrP. The test was replicated three times at 60 s intervals of the same level of pain by participants. The results of the threshold of active MTrP were recorded, and the mean of the PPT of MTrPs of upper trapezius muscles was calculated.

#### 2.3.3. Tissue Hardness of Upper Trapezius Muscles

Soft tissue stiffness was measured by using a tissue hardness meter (OE-220, ITO Co., Ltd., Tokyo, Japan) and applied in clinical studies recently [17–19]. The physician placed the metal probe of the tissue hardness meter vertically onto the MTrP of the upper trapezius muscle and pressurized by 1 mm/s. The test was finished when reaching a 10 mm measurement distance. The average of three readings was used for tissue hardness analysis. Every test had a 1 min intermission.

#### 2.3.4. Neck Range of Motion

**Figure 5:**
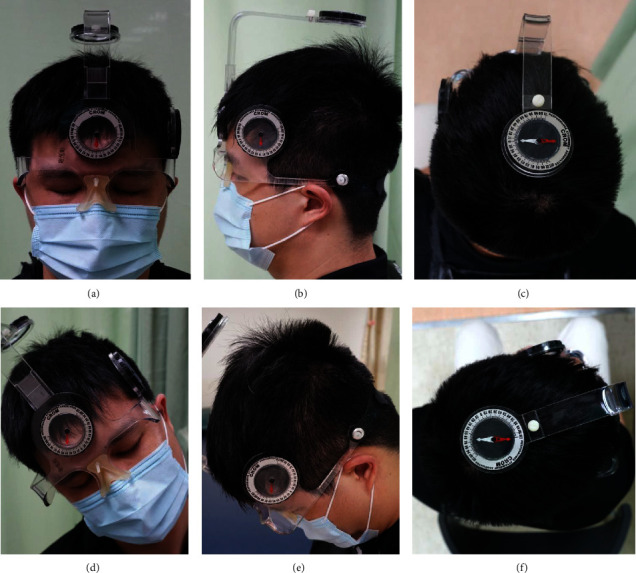
Neck range of motion assessment. Participants were asked to wear the Cervical Range of Motion (CROM) instrument. Zeroing the gravity inclinometer at the front (a), at the lateral (b), and on the top (c) of the device before assessment. The red arrow of three inclinometers indicated the angle of zero. The angle change was measured when participants did the action of neck movement, that is, right-side bending (d), flexion (e), and right rotation (f).

Cervical Range of Motion (CROM) instrument (Performance Attainment Associates, 958 Lydia Drive, Roseville, MN 55113) was used to assess NROM [20–22]. A gravity inclinometer was used to measure the NROM when participants performed the actions of flexion, extension, left rotation, right rotation, left-side bending, and right-side bending. The three inclinometers on the top, at the front, and at the lateral of the device indicated the 3D angle of neck motion (Figures 5(a)–5(f)).

#### 2.3.5. Neck Disability Index

Neck disability index (NDI) was modified from the Oswestry Low Back Pain Index [23], and it is the most popular self-rated neck disability instrument due to neck pain [24]. Each of the 10 items was scored from 0 to 5 to achieve a sum of 50 scores. Participants finished the questionnaire before day 1 experiment and on day 8 and day 15 follow-up. The scoring sum below 5 indicated no activity limitation. The sum of 5–14 indicated a mild disability. The sum of 15–24 indicated an intermediate disability. The sum of 25–34 indicated a severe disability. The sum over 34 indicated complete activity limitation.

#### 2.3.6. Pittsburgh Sleep Quality Index

PSQI is the most effective tool to evaluate sleep quality in adults [25, 26]. The questions comprised subjective sleep quality, sleep latency, sleep duration, habitual sleep efficiency, step disturbance, use of sleeping medication, and daytime dysfunction during the past month. In each item, a score of 0 indicated no difficulty, whereas a score of 3 indicated severe difficulty. The global score of total items yielded a range from 0 to 21. A global score of 5 or more was considered poor sleep quality.

### 2.4. Statistical Analysis

Statistically significant differences (*P* < 0.05) among the results were calculated by using Statistical Package for Social Science (SPSS 18.0) for Windows. All data were expressed as mean ± standard deviation (SD). Baseline characteristics analysis of age, sex, VAS, PTT, TH, NROM, NDI, and PSQI was conducted via Student's *t*-test. For inferential statistics, the within-group analysis of all variables was conducted by paired sample *t*-test, whereas the between-group analysis of the variables was conducted by independent two-sample *t*-test.

## 3. Results

### 3.1. Baseline Characteristics of Two Groups of Participants in the Study

The baseline characteristics and outcome measurements of the two groups are shown in Table 1. The mean of age was 52.73 ± 9.81 years for the FSN group and 52.16 ± 16.10 years for the TENS group, without significant differences (*P*=0.870). The number of female participants was higher than the number of male participants in both groups (male: female, 10 : 20 for FSN; 8 : 22 for TENS). The affected side of the neck in the left and right sides were 17 and 13 participants for FSN and 14 and 16 participants for TENS, respectively. The VAS value was not significantly different in the FSN group compared with that in the TENS group (5.95 ± 1.36 for FSN and 6.71 ± 1.80 for TENS, *P*=0.069). TH was similar in the two groups (56.75 ± 8.03 for FSN; 56.80 ± 9.49 for TENS, *P*=0.985). No significant difference was observed in PPT (37.40 ± 5.11 for FSN; 39.31 ± 6.63 for TENS, *P*=0.216) and NROM, including flexion, extension, left rotation, right rotation, left-side bending, and right-side bending in the two groups. Outcome assessments by questionnaire of NDI and PSQI showed no significant difference. No significant difference was found in all baseline values between the two groups. These results provide a well-randomized prospective study for further investigation.

### 3.2. FSN Treatment Reduces Chronic Neck Pain and Tissue Hardness Immediately

**Figure 6:**
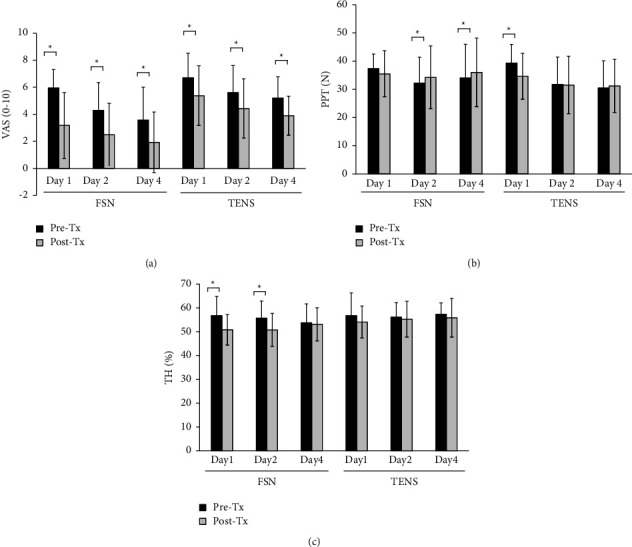
Comparison the immediate effects of the two groups. The pretreatment and posttreatment value of VAS (a), PPT (b), and TH (c) was measured in three treatment sessions in both groups. Asterisks (^*∗*^) showed the *P* < 0.05. VAS: visual analog scale, PPT: pressure pain threshold, and TH: tissue hardness.

To understand the immediate effect of FSN on chronic neck pain, we evaluated the VAS, PPT, and TH before and after treatment. The data are shown in Table 2 and Figure 6. VAS (pre-Tx: 5.95 ± 1.36 vs. post-Tx: 3.18 ± 2.43, *P* < 0.001) and TH (pre-Tx: 56.75 ± 8.03 vs. post-Tx: 50.80 ± 6.38, *P* < 0.001) significantly improved in the FSN group on day 1, except PPT (pre-Tx: 37.40 ± 5.11 vs. post-Tx: 35.50 ± 8.19, *P*=0.117). For the TENS group, VAS (pre-Tx: 6.71 ± 1.80 vs. post-Tx: 5.38 ± 2.21, *P* < 0.001) and PTT (pre-Tx: 39.31 ± 6.63 vs. post-Tx: 34.65 ± 8.11, *P* < 0.001) also significantly improved, except TH (pre-Tx: 56.80 ± 9.49 vs. post-Tx:54.06 ± 6.71). In the difference comparison (Table 2), FSN was more effective in pain relief (−2.76 ± 1.68 for FSN, −1.33 ± 1.02 for TENS, *P* < 0.001), not in PPT (−1.89 ± 6.42 for FSN, −4.66 ± 4.60 for TENS, *P*=0.060) and TH (−5.95 ± 7.72 for FSN, −2.73 ± 8.22 for TENS, *P*=0.124) compared with TENS. On day 2, VAS (pre-Tx: 4.30 ± 2.05, post-Tx: 2.50 ± 2.31, *P* < 0.001), PPT (pre-Tx: 32.21 ± 9.19, post-Tx: 34.27 ± 11.13, *P*=0.006), and TH (pre-Tx: 55.75 ± 7.12, post-Tx: 50.78 ± 6.86, *P*=0.001) significantly improved in the FSN group. However, TENS only significantly improved VAS (pre-Tx: 5.61 ± 2.00, post-Tx: 4.43 ± 2.19, *P* < 0.001), not PPT (*P*=0.738) and TH (*P*=0.596), on day 2. The comparison of differences revealed that FSN was more effective on VAS (FSN: −1.80 ± 1.37, TENS: −1.18 ± 0.79, *P*=0.038) and PPT (FSN: 2.06 ± 3.80, TENS: −0.20 ± 3.29, *P*=0.017) and TH (FSN: −4.97 ± 6.00, TENS: −0.87 ± 8.95, *P*=0.042) than TENS after the second course of treatment.

After an intermission of 1 day, the day 4 evaluation results showed that FSN still had a significant effect on VAS (pre-Tx: 3.58 ± 2.42, post-Tx: 1.93 ± 2.24, *P* < 0.001) and PPT (pre-Tx: 34.08 ± 11.95, post-Tx: 36.03 ± 12.16, *P*=0.023) but not on TH (*P*=0.705). Only a significant effect on VAS (pre-Tx: 5.20 ± 1.58, post-Tx: 3.90 ± 1.44, *P* < 0.001) was observed in the TENS group. However, the difference between FSN and TENS in VAS, PPT, and TH was not significant.

### 3.3. Short-Term and Long-Term Effects of FSN on Pain Relief

**Figure 7:**
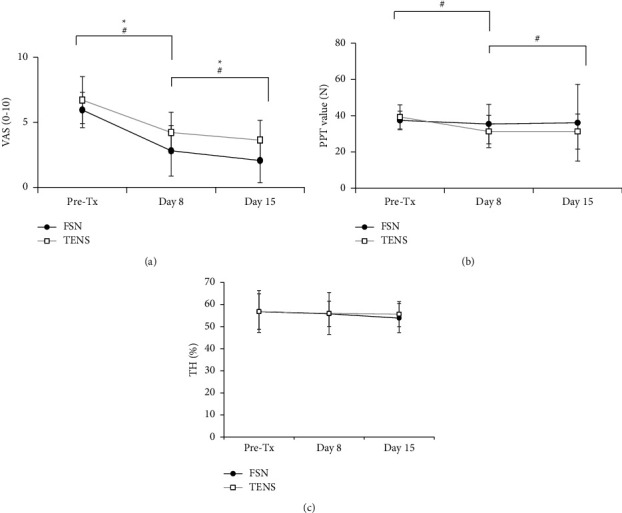
Comparison the short-term and long-term effects of the two groups. The value of VAS (a), PPT (b), and TH (c) was measured on day 1 before treatment and followed up to day 8 and day 15 in both groups. Asterisks (^*∗*^) and hashtag (^#^) showed the *P* < 0.05 in FSN or TENS group, respectively. VAS: visual analog scale, PPT: pressure pain threshold, TH: tissue hardness, PFG: pain free grip, FSN: Fu's subcutaneous needling, and TENS: transcutaneous electrical nerve stimulation.

In VAS test, both FSN and TENS demonstrated a decrease in pain scale on day 8 and day 15 (*P* < 0.001 in all tests; Table 3 and Figure 7). Only TENS decreased PPT on day 8 and day 15 (*P* < 0.001), whereas FSN had no significant effect on PTT. In addition, no significant decrease in TH was found in both groups on day 8 and day 15. Interestingly, TENS had a more significant decrease of PPT compared with FSN on day 8 and day 15 (day 8: −1.27 ± 11.67 for FSN, −8.02 ± 8.51 for TENS, *P*=0.017; day 15: −0.48 ± 12.98 for FSN, −8.02 ± 9.17 for TENS, *P*=0.020; Table 3).

### 3.4. FSN Improved Neck Disability, Mobility, and Sleep Quality

**Figure 8:**
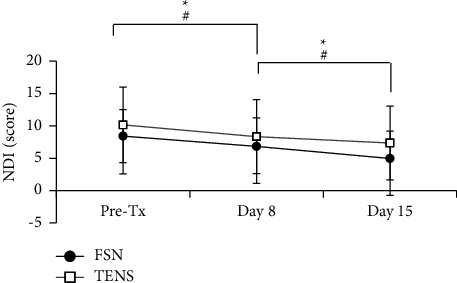
Comparison of the short-term and long-term effects on NDI. The score of NDI was measured on day 1 pre-treatment and followed up to day 8 and day 15 in both groups. Asterisks (^*∗*^) and hashtag (^#^) showed the *P* < 0.05 in FSN or TENS group, respectively. FSN: Fu's subcutaneous needling, TENS: transcutaneous electrical nerve stimulation; NDI: neck disability index.

Chronic neck pain commonly induces limitations of neck range of motion, followed by poor sleep quality. To estimate the effect of FSN on neck mobility, we analyzed the subjective questionnaire of NDI and objective NROM. The data in Table 4 and Figure 8 showed that the score of NDI improved by FSN from 8.43 ± 4.09 on day 1 pre-Tx to 6.83 ± 4.39 on day 8 (*P*=0.010), and the effectiveness was sustained to day 15 (4.96 ± 4.23, *P* < 0.001). The effectiveness of TENS on NDI was from 10.16 ± 5.84 on day 1 pre-Tx to 8.33 ± 5.73 on day 8, *P*=0.036; and to 7.36 ± 5.70 on day 15, *P*=0.001. The two groups showed significant effects on short-term and long-term NDI assessment.

To understand further the effect of FSN on neck mobility, NROM was measured during pre- and post-treatment. Both FSN and TENS had benefits on neck motion upon treatment at days 1 and 2, except left rotation in the TENS group (Table 5). After an intermission of 1 day, FSN had benefits on the action of extension, right rotation, right rotation and left/right-side bending on day 4 treatment, whereas TENS had benefits on the actions of flexion, extension, left rotation, and right-side bending (Table 5). On day 8 and day 15 follow-up, both FSN and TENS had benefits on all active neck motion, except TENS for neck extension (Table 6).

Improvement in sleep quality is an important outcome to assess the effectiveness of therapy. The results of the self-reported PSQI questionnaire indicated that FSN was beneficial for participants to achieve better sleep quality on day 15 follow-up compared with TENS (in the FSN group, from 10.67 ± 3.05 on day 1 pre-Tx to 10.43 ± 2.69 on day 8, *P*=0.504, and to 9.93 ± 2.74 on day 15, *P*=0.030; in the TENS group, from 10.80 ± 3.81 in pre-Tx day 1 to 10.26 ± 3.67 on day 8, *P*=0.290, and to 10.26 ± 3.18 on day 15, *P*=0.252; Table 7).

## 4. Discussion

To our knowledge, this study was the first to uncover and demonstrate the effectiveness of FSN in chronic neck pain treatment. We examined the improvement in VAS, PPT, TH, NROM, and outcome measurement of NDI and PSQI after treatment in patients with chronic neck pain. The results of this clinical trial showed that FSN had significant benefits on VAS, NDI, and sleep quality at 15 days follow-up compared with TENS treatment.

TENS is a widely used modality in clinical practice for chronic neck pain with advantages of non-invasive, safe, and immediate effects on pain relief [27]. It is based on electrodes attached to the pain area or acupoints for current transmission. This action stimulates non-nociceptive neuron fibers to block pain transmission in accordance with gate control theory [28]. However, its effectiveness is controversial [29, 30]. A meta-analysis of clinical studies showed insufficient evidence regarding treatment with TENS in patients with chronic neck pain [30]. Nevertheless, we observed immediate pain reduction after TENS treatment on day 1, day 2, and day 4 (Table 2), and TENS-reduced pain effect was sustained to day 8 and day 15 follow-up (Table 3).

Purpose-based acupoints are used for the treatment of various symptoms in ancient acupuncture theories. Needling on different acupoints produces distinct effects. For example, acupuncture at SI 3 (*Houxi*) and TE 3 (*Zhongzhu*) is effective for acute neck pain caused by stiff neck or cervical spondylosis [31, 32]. In chronic neck pain treatments, TE 5 (*Waiguan*) and LI 11 (*Quchi*) are commonly used in acupuncture and TENS [7]. However, the needling points of FSN are on the midpoint of the extensor muscle of forearm, not on the acupoints or MTrPs, different from conventional acupuncture in our study. A treatment strategy focusing on the upper trapezius muscle may be the key to eliminating the condition. For example, muscle energy technique and ischemic compression technique on upper trapezius active MTrPs have a short-term effect on pain relief in patients with nonspecific neck pain [33, 34]. In this study, we observed the remote effect of FSN; needling the distal location from the MTrP area led to pain relief and a sustained effect on neck motion and sleep quality (Tables 2, 3, and 5–7). The mechanism may be that needling the myofascial layer triggered the signal transduction of connective tissue to relax the tightened muscles, that is, upper trapezius muscle for chronic neck pain. By combining the swaying and reperfusion approach, FSN effectively relieved neck pain and promoted the remission of the limitation of NROM. Swaying movement of the FSN in the subcutaneous layer released the muscular tension of affected muscles, resulting in pain relief and decreased tissue hardness immediately. The reperfusion approach rapidly restored blood flow, and re-congestion in damaged muscle resulted in accelerated tissue repair. The resistance action of contraction of the upper trapezius muscle helped with the recovery of the disorder.

Tissue hardness or stiffness is the ability of muscle to resist deformation when doing activities. Increase in tissue hardness implies that someone requires more power and energy to respond to the activity of the agonist and antagonistic muscle. The difference between the affected side and the normal side of neck and shoulders can break the coordination. Our data supported the improvement effect of FSN on TH in the first 2 days of treatment. With improvement in neck pain and tissue hardness, FSN could improve neck disability (Table 5). Most people have a problem of poor posture due to looking downward to work or using their cellphone or personal computer for a long period. Immense stress makes people have an involuntary shrug that can cause neck pain. Exercises can improve one's posture to correct positions that prevent neck pain or intervertebral disc herniation [35]. Self-training of the neck muscle is recommended for patients with chronic neck pain and effectively reduces neck pain [36]. If patients are treated with FSN and combine exercise practice, they may stop the disease process of neck pain and the symptoms. The efficacy of combination treatments needs more investigation.

Needle therapy, including dry needling, for chronic neck pain is usually used with more than one filament needle to needle into the MTrPs directly or nearby areas; however, the effectiveness of dry needling on chronic neck pain is equivocal, recently reported by a long-term follow-up trial [37] and a meta-analysis study [38]. Furthermore, given that most people are high responders to needle pain [39], remote therapy and needle-less methods are the better options for health care. Remote injection with anesthetics has been demonstrated to be an effective treatment for chronic neck pain [40]. The analgesic effects of intramuscular lidocaine injection act on voltage-gated sodium channels to block nerve conduction and sensation in the peripheral nervous system [41]. In our study, we observed that remote FSN on chronic neck pain benefitted pain relief. FSN is suitable for patients who fear needle pain by using the disposable needle insertion away from MTrPs, and this method involves minimal pain.

The FSN needle is inserted into the subcutaneous layer, which contains adipose tissue, connective tissues, and numerous vascular and neural networks. Half a century ago, Boguslaw Lipinski reported that the potential mechanism of acupuncture relies on the piezoelectric effect from connective tissues [42]. Furthermore, the neural pathway is the mechanism involved in acupuncture [43], which is applied to nervous system diseases [44]. The effect of acupuncture was blocked by local anesthetic injection in a rat model [45], indicating that peripheral sensory nerves are involved in the action of acupuncture. The mechanical connective tissue reaction instead of neural mechanism in FSN treatment was first investigated in a rabbit model in 2012 [46]. Monitoring the endplate noise from rabbit myofascial trigger spots (MTrSs) with FSN intervention demonstrated that FSN to MTrSs of distal ipsilateral gastrocnemius muscle can initially increase the irritability of MTrS in proximal biceps femoris muscle, followed by a suppression effect after cessation of needling, but these observations were not found in the contralateral side [46]. This hypothesis was also supported in the study of Langevin and her colleagues [47], who hypothesized that mechanical coupling between the needle and connective tissue with winding of tissue around the needle during needle rotation transmits a mechanical signal to connective tissue cells that may explain local and remote, as well as long-term, effects of acupuncture. Unlike Hsieh and her colleagues' animal study of dry needling [48], the mechanism for the effectiveness of dry needling and acupuncture to MTrP-induced disorders was related to an intact neural network. The effectiveness of remote FSN may go through the piezoelectricity and mechanical connective tissue reaction instead of neural mechanism.

### 4.1. Limitations

This study had some limitations. First, patients with chronic neck pain usually have an accompanying disability such as limited neck motion and poor sleep quality. The disorder is not restricted to elders only; up to 67% of the young population (aged 18–29 years) have had a 12-month prevalence of chronic neck pain [49]. Young people often recover more quickly than elders. In our study, only four young people were recruited in the TENS group with a smaller VAS score of 5 and 6. This may not influence the effect of FSN or TENS on chronic neck pain in this study. Second, patients with the TENS intervention in this study comprised the control group; however, it was not a real placebo group compared with the FSN group. A sham FSN design is needed to be an ideal placebo group when compared with the FSN group to evaluate the treatment's effectiveness. Sham FSN may be designed by intervention with a swaying or reperfusion approach alone, or without both procedures. As expected, the sham FSN may not provide any improvement on chronic neck pain symptoms, but neck pain decreased while the FSN needle was penetrated into the skin. However, choosing an ineffectual treatment was impossible for subjects in this study. We were obliged to take TENS as the control group to compare the effectiveness of FSN on chronic neck pain. The small sample size was another limitation of this study, which resulted in a small statistical power. Small sample sizes make some results inconclusive. For example, a significant improvement was observed for neck disability in the FSN and TENS groups and sleep quality in the FSN group only (Table 4, Figure 8, and Table 7). However, we could not prove the beneficial effects of FSN compared with TENS treatment via difference analysis on day 8 and day 15 follow-up, even if the difference was greater in the FSN group than in the TENS group (all *P* > 0.05), Supplementary Table 1 and Supplementary Table 2. Future research is required to establish a large population involving other institutes to amplify the statistical power and reach conclusive results.

## 5. Conclusions

This study is the first to investigate FSN treatment of chronic neck pain with scientific evidence by using several objective evaluation tools in a clinical setting. FSN could not only relieve neck pain but also it improved the PPT and TH. Notably, FSN significantly improved neck disability and enhanced sleep quality.

## Figures and Tables

**Table 1:** Baseline characteristics of participants in two groups.

Characteristic	FSN	TENS	*P* value
Number	30	30	
Age (year)	52.73 ± 9.81	52.16 ± 16.10	0.870
Gender, male/female, number (%)	10/20 (33%/67%)	8/22 (27%/73%)	
ASON, left/right, number (%)	17/13 (57%/43%)	14/16 (47%/53%)	
VAS (0–10)	5.95 ± 1.36	6.71 ± 1.80	0.069
PPT (N)	37.40 ± 5.11	39.31 ± 6.63	0.216
TH (%)	56.75 ± 8.03	56.80 ± 9.49	0.985

NROM index (degrees)			
Flexion	49.80 ± 12.47	47.60 ± 11.97	0.489
Extension	49.43 ± 14.46	53.13 ± 12.71	0.297
Left rotation	54.13 ± 11.39	54.83 ± 12.84	0.824
Right rotation	57.56 ± 12.21	56.96 ± 9.75	0.834
Left side bending	41.03 ± 10.75	40.90 ± 11.16	0.963
Right side bending	37.50 ± 8.61	35.30 ± 10.12	0.368
NDI (0–50)	8.43 ± 4.09	10.16 ± 5.84	0.189
PSQI (0–21)	10.67 ± 3.05	10.80 ± 3.81	0.882

Data were expressed as mean ± SD; *P* value was tested with an independent two-sample *t*-test. ASON: the affected side of neck; FSN: Fu's subcutaneous needling; TENS: transcutaneous electrical nerve stimulation; VAS: visual analog scale; PPT: pain pressure threshold; TH: tissue hardness of muscle; NROM: neck range of motion; NDI: neck disability index; PSQI: Pittsburgh sleep quality index.

**Table 2:** Immediate effects of FSN and TENS groups on VAS, PPT, and TH.

	FSN			TENS			Difference		
	Pre-tx	Post-tx	*P* ^*a*^	Pre-tx	Post-tx	*P* ^*a*^	FSN	TENS	*P* ^*b*^
Day 1									
VAS (1–10)	5.95 ± 1.36	3.18 ± 2.43	<0.001	6.71 ± 1.80	5.38 ± 2.21	<0.001	−2.76 ± 1.68	−1.33 ± 1.02	<0.001
PPT (N)	37.40 ± 5.11	35.50 ± 8.19	0.117	39.31 ± 6.63	34.65 ± 8.11	<0.001	−1.89 ± 6.42	−4.66 ± 4.60	0.060
TH (%)	56.75 ± 8.03	50.80 ± 6.38	<0.001	56.80 ± 9.49	54.06 ± 6.71	0.079	−5.95 ± 7.72	−2.73 ± 8.22	0.124

Day 2									
VAS (1–10)	4.30 ± 2.05	2.50 ± 2.31	<0.001	5.61 ± 2.00	4.43 ± 2.19	<0.001	−1.80 ± 1.37	−1.18 ± 0.79	0.038
PPT (N)	32.21 ± 9.19	34.27 ± 11.13	0.006	31.77 ± 9.70	31.56 ± 10.19	0.738	2.06 ± 3.80	−0.20 ± 3.29	0.017
TH (%)	55.75 ± 7.12	50.78 ± 6.86	<0.001	56.14 ± 6.03	55.26 ± 7.49	0.596	−4.97 ± 6.00	−0.87 ± 8.95	0.042

Day 4									
AS (1–10)	3.58 ± 2.42	1.93 ± 2.24	<0.001	5.20 ± 1.58	3.90 ± 1.44	<0.001	−1.65 ± 1.15	−1.30 ± 0.74	0.169
PPT (N)	34.08 ± 11.95	36.03 ± 12.16	0.023	30.54 ± 9.55	31.25 ± 9.38	0.107	1.95 ± 4.46	0.70 ± 2.31	0.178
TH (%)	53.74 ± 7.95	53.12 ± 6.95	0.705	57.29 ± 4.81	55.86 ± 8.13	0.326	−0.62 ± 8.89	−1.43 ± 7.86	0.709

Data were expressed as mean ± SD.*P*^*a*^ value was tested with a paired sample *t*-test.*P*^*b*^ value was tested with an independent two-sample *t*-test. FSN: Fu's subcutaneous needling; TENS: transcutaneous electrical nerve stimulation; VAS: visual analog scale; PPT: pain pressure threshold; TH: tissue hardness of muscle.

**Table 3:** Short-term and long-term effects of FSN and TENS groups on VAS, PPT, and TH.

	Pre-tx	Day 8	*P* ^*a*^	Difference	*P* ^*c*^	Day 15	*P* ^*b*^	Difference	*P* ^*c*^
VAS (0–10)									
FSN	5.95 ± 1.36	2.81 ± 1.94	<0.001	−3.16 ± 1.78	0.204	2.06 ± 1.70	<0.001	−3.95 ± 1.61	0.093
TENS	6.71 ± 1.80	4.21 ± 1.57	<0.001	−2.50 ± 2.01		3.63 ± 1.52	<0.001	−3.08 ± 1.95	

PPT (N)									
FSN	37.40 ± 5.11	35.43 ± 10.86	0.589	−1.27 ± 11.67	0.017	36.09 ± 12.14	0.158	−0.48 ± 12.98	0.020
TENS	39.31 ± 6.63	31.28 ± 8.88	<0.001	−8.02 ± 8.51		31.28 ± 9.68	<0.001	−8.02 ± 9.17	

TH (%)									
FSN	56.75 ± 8.03	55.77 ± 5.73	0.310	−1.47 ± 9.42	0.957	53.83 ± 6.56	0.567	−3.27 ± 10.53	0.500
TENS	56.80 ± 9.49	55.95 ± 9.49	0.594	−0.85 ± 8.64		55.62 ± 5.64	0.462	−1.18 ± 8.66	

Data were expressed as mean ± SD; *P* value was tested with an independent two-sample *t*-test. ^*a*^Compares the value in pre-Tx and on day 8 of FSN or TENS group. ^*b*^Compares the value on day 8 and on day 15 of FSN or TENS group. ^*c*^compares the value of difference between FSN and TENS group. FSN: Fu's subcutaneous needling; TENS: transcutaneous electrical nerve stimulation; VAS: visual analog scale; PPT: pain pressure threshold; TH: Tissue hardness of muscle.

**Table 4:** Short-term and long-term effects of FSN and TENS groups on NDI.

	Pre-tx on day 1	Day 8	*P* ^*a*^	Day 15	*P* ^*b*^
FSN	8.43 ± 4.09	6.83 ± 4.39	0.010	4.96 ± 4.23	<0.001
TENS	10.16 ± 5.84	8.33 ± 5.73	0.036	7.36 ± 5.70	0.001

Data were expressed as mean ± SD; *P* value was tested with a paired sample *t*-test. ^*a*^Compares the value in pre-Tx and on day 8 of FSN or TENS group. ^*b*^Compares the value on day 8 and on day 15 of FSN or TENS group. FSN: Fu's subcutaneous needling; TENS: transcutaneous electrical nerve stimulation; NDI: neck disability index.

**Table 5:** Immediate effects of FSN and TENS groups on NROM.

	FSN			TENS		
	Pre-tx	Post-tx	*P*	Pre-tx	Post-tx	*P*
Day 1						
Flexion	49.80 ± 12.47	55.50 ± 11.15	<0.001^*∗*^	47.60 ± 11.97	51.16 ± 11.05	0.001^*∗*^
Extension	49.43 ± 14.46	54.76 ± 12.24	<0.001^*∗*^	53.13 ± 12.71	56.33 ± 14.03	0.043^*∗*^
Left rotation	54.13 ± 11.39	60.10 ± 9.72	<0.001^*∗*^	54.83 ± 12.84	57.56 ± 10.40	0.052
Right rotation	57.56 ± 12.21	63.30 ± 10.48	<0.001^*∗*^	56.96 ± 9.75	60.26 ± 9.81	0.002^*∗*^
Left side bending	41.03 ± 10.75	44.13 ± 9.79	0.034^*∗*^	40.90 ± 11.16	44.33 ± 10.90	0.044^*∗*^
Right side bending	37.50 ± 8.61	42.53 ± 6.86	<0.001^*∗*^	35.30 ± 10.12	39.66 ± 8.78	0.001^*∗*^

Day 2						
Flexion	54.23 ± 10.83	57.80 ± 11.04	0.001^*∗*^	52.33 ± 11.67	54.46 ± 11.61	0.030^*∗*^
Extension	52.46 ± 12.55	57.20 ± 11.80	0.001^*∗*^	52.83 ± 12.16	56.56 ± 11.70	<0.001^*∗*^
Left rotation	58.26 ± 9.08	60.76 ± 9.55	0.034^*∗*^	59.50 ± 11.38	61.40 ± 11.06	0.040^*∗*^
Right rotation	60.10 ± 11.05	62.73 ± 9.64	0.040^*∗*^	59.00 ± 9.25	63.06 ± 8.43	0.002^*∗*^
Left side bending	43.26 ± 9.77	47.53 ± 8.57	0.010^*∗*^	41.80 ± 10.99	45.06 ± 9.06	0.005^*∗*^
Right side bending	40.13 ± 7.17	44.30 ± 7.44	<0.001^*∗*^	38.00 ± 9.72	41.70 ± 9.02	<0.001^*∗*^

Day 4						
Flexion	57.16 ± 11.52	58.70 ± 11.22	0.115	53.20 ± 12.31	55.36 ± 11.11	0.048^*∗*^
Extension	55.06 ± 10.57	58.70 ± 9.70	<0.001^*∗*^	56.16 ± 11.34	59.16 ± 11.06	0.002^*∗*^
Left rotation	57.63 ± 10.41	59.76 ± 8.69	0.077	59.63 ± 9.89	62.30 ± 8.17	0.003^*∗*^
Right rotation	61.33 ± 11.25	65.63 ± 9.29	0.016^*∗*^	62.30 ± 8.81	63.30 ± 9.06	0.187
Left side bending	44.03 ± 9.17	48.16 ± 8.95	<0.001^*∗*^	45.03 ± 10.37	47.30 ± 9.27	0.077
Right side bending	44.16 ± 9.50	47.10 ± 8.46	0.011^*∗*^	40.80 ± 8.41	43.76 ± 8.63	0.001^*∗*^

Data were expressed as mean ± SD; *P* value was tested with a paired sample *t*-test. FSN: Fu's subcutaneous needling; TENS: transcutaneous electrical nerve stimulation; NROM: neck range of motion. Asterisks (^*∗*^) showed the *P* < 0.05 in FSN or TENS group, respectively.

**Table 6:** Short-term and long-term effects of FSN and TENS groups on NROM.

	Pre-tx	Day 8	*P* ^*a*^	Day 15	*P* ^*b*^
Flexion					
FSN	49.80 ± 12.47	56.30 ± 10.50	0.004^*∗*^	58.50 ± 10.10	<0.001^*∗*^
TENS	47.60 ± 11.97	54.96 ± 9.96	<0.001^*∗*^	57.26 ± 10.94	<0.001^*∗*^
Extension					
FSN	49.43 ± 14.46	56.30 ± 10.50	<0.001^*∗*^	57.20 ± 11.01	<0.001^*∗*^
ENS	53.13 ± 12.71	56.00 ± 9.72	0.066	56.16 ± 11.42	0.058
Left rotation					
FSN	54.13 ± 11.39	59.30 ± 9.62	0.002^*∗*^	61.83 ± 9.12	<0.001^*∗*^
TENS	54.83 ± 12.84	61.50 ± 7.96	0.001^*∗*^	62.53 ± 7.40	0.002^*∗*^
Right rotation					
FSN	57.56 ± 12.21	62.60 ± 10.26	0.024^*∗*^	64.70 ± 8.23	0.001^*∗*^
TENS	56.96 ± 9.75	63.86 ± 9.77	<0.001^*∗*^	63.56 ± 9.79	<0.001^*∗*^
Left side bending					
FSN	41.03 ± 10.75	44.76 ± 7.48	0.013^*∗*^	47.06 ± 8.37	0.002^*∗*^
TENS	40.90 ± 11.16	47.33 ± 9.21	<0.001^*∗*^	47.30 ± 11.48	0.001^*∗*^
Right side bending					
FSN	37.50 ± 8.61	43.80 ± 7.09	<0.001^*∗*^	45.66 ± 8.38	<0.001^*∗*^
TENS	35.30 ± 10.12	42.33 ± 8.35	<0.001^*∗*^	43.83 ± 8.53	<0.001^*∗*^

Data were expressed as mean ± SD; *P* value was tested with a paired sample *t*-test. ^*a*^Compares the value in pre-Tx and on day 8 of FSN or TENS group. ^*b*^Compares the value on day 8 and on day 15 of FSN or TENS group. Asterisks (^*∗*^) showed the *P* < 0.05 in FSN or TENS group, respectively. FSN: Fu's subcutaneous needling; TENS: transcutaneous electrical nerve stimulation; NROM: neck range of motion.

**Table 7:** Effectiveness of FSN and TENS groups on sleep quality via self-reported PSQI questionnaire.

	Pre-tx on day 1	Day 8	*P* ^*a*^	Day 15	*P* ^*b*^
FSN	10.67 ± 3.05	10.43 ± 2.69	0.504	9.93 ± 2.74	0.030^*∗*^
TENS	10.80 ± 3.81	10.26 ± 3.67	0.290	10.26 ± 3.18	0.252

Data were expressed as mean ± SD; *P* value was tested with a paired sample *t*-test.^*a*^ Compares the value in pre-Tx and on day 8 of FSN or TENS group. ^*b*^Compares the value on day 8 and on day 15 of FSN or TENS group. Asterisks (^*∗*^) showed the *P* < 0.05. FSN: Fu's subcutaneous needling; TENS: transcutaneous electrical nerve stimulation; PSQI: Pittsburgh sleep quality index.

## Data Availability

The data that support the findings of this study are available from the corresponding author, upon reasonable request.

## Supplementary Materials

Supplementary Materials
*Supplementary Table 1.* Difference comparison of NDI in two groups. *Supplementary Table 2.* Difference comparison of PSQI in two groups.

## References

[1] Goode A. P., Freburger J., Carey T. (2010). Prevalence, practice patterns, and evidence for chronic neck pain. *Arthritis Care & Research*.

[2] Safiri S., Kolahi A. A., Hoy D. (2020). Global, regional, and national burden of neck pain in the general population, 1990-2017: systematic analysis of the global burden of disease study 2017. *BMJ*.

[3] Binder A. I. (2008). Neck pain. *BMJ Clinical Evidence*.

[4] Fejer R., Kyvik K. O., Hartvigsen J. (2006). The prevalence of neck pain in the world population: a systematic critical review of the literature. *European Spine Journal*.

[5] Amaechi O., Huffman M. M., Featherstone K. (2021). Pharmacologic therapy for acute pain. *American Family Physician*.

[6] Chiu T. T., Hui-Chan C. W., Cheing G. (2005). A randomized clinical trial of TENS and exercise for patients with chronic neck pain. *Clinical Rehabilitation*.

[7] Chou L. W., Hsieh Y. L., Chen H. S., Hong C. Z., Kao M. J., Han T. I. (2011). Remote therapeutic effectiveness of acupuncture in treating myofascial trigger point of the upper trapezius muscle. *American Journal of Physical Medicine & Rehabilitation*.

[8] Cerezo-Tellez E., Torres-Lacomba M., Fuentes-Gallardo I. (2016). Effectiveness of dry needling for chronic nonspecific neck pain: a randomized, single-blinded, clinical trial. *Pain*.

[9] Fu Z. H. (2016). *The Foundation of Fu’s Subcutaneous Needling*.

[10] Dommerholt J., Fernandez de las Penas C. (2018). *Trigger Point Dry Needling: An Evidence and Clinical-Based Approach*.

[11] Huang C. H., Lin C. Y., Sun M. F., Fu Z., Chou L. W. (2022). Efficacy of fu’s subcutaneous needling on myofascial trigger points for lateral epicondylalgia: a randomized control trial. *Evidence-Based Complementary and Alternative Medicine*.

[12] (1986). Classification of chronic pain. Descriptions of chronic pain syndromes and definitions of pain terms. prepared by the international association for the study of pain, subcommittee on taxonomy. *Pain-Supplement*.

[13] Reed M. D., Van Nostran W. (2014). Assessing pain intensity with the visual analog scale: a plea for uniformity. *The Journal of Clinical Pharmacology*.

[14] Haefeli M., Elfering A. (2006). Pain assessment. *European Spine Journal*.

[15] Fischer A. A. (1986). Pressure threshold meter: its use for quantification of tender spots. *Archives of Physical Medicine and Rehabilitation*.

[16] Fischer A. A. (1987). Pressure algometry over normal muscles. Standard values, validity and reproducibility of pressure threshold. *Pain*.

[17] Cheng Z., Chen Z., Xie F. (2021). Efficacy of Yijinjing combined with Tuina for patients with non-specific chronic neck pain: study protocol for a randomized controlled trial. *Trials*.

[18] Zheng Z., Wang J., Gao Q. (2012). Therapeutic evaluation of lumbar tender point deep massage for chronic non-specific low back pain. *Journal of Traditional Chinese Medicine*.

[19] Wamontree P., Kanchanakhan N., Eungpinichpong W., Jeensawek A. (2015). Effects of traditional Thai self-massage using a Wilai massage stick(TM) versus ibuprofen in patients with upper back pain associated with myofascial trigger points: a randomized controlled trial. *Journal of Physical Therapy Science*.

[20] Mejuto-Vazquez M. J., Salom-Moreno J., Ortega-Santiago R., Truyols-Dominguez S., Fernandez-de-las-Penas C. (2014). Short-term changes in neck pain, widespread pressure pain sensitivity, and cervical range of motion after the application of trigger point dry needling in patients with acute mechanical neck pain: a randomized clinical trial. *Journal of Orthopaedic & Sports Physical Therapy*.

[21] Carvalho G. F., Chaves T. C., Goncalves M. C. (2014). Comparison between neck pain disability and cervical range of motion in patients with episodic and chronic migraine: a cross-sectional study. *Journal of Manipulative and Physiological Therapeutics*.

[22] Inokuchi H., Tojima M., Mano H., Ishikawa Y., Ogata N., Haga N. (2015). Neck range of motion measurements using a new three-dimensional motion analysis system: validity and repeatability. *European Spine Journal*.

[23] Vernon H., Mior S. (1991). The neck disability index: a study of reliability and validity. *Journal of Manipulative and Physiological Therapeutics*.

[24] MacDermid J. C., Walton D. M., Avery S. (2009). Measurement properties of the neck disability index: a systematic review. *Journal of Orthopaedic & Sports Physical Therapy*.

[25] Buysse D. J., Reynolds C. F., Monk T. H., Berman S. R., Kupfer D. J. (1989). The Pittsburgh Sleep Quality Index: a new instrument for psychiatric practice and research. *Psychiatry Research*.

[26] Knutson K. L., Rathouz P. J., Yan L. L., Liu K., Lauderdale D. S. (2006). Stability of the Pittsburgh sleep quality index and the epworth sleepiness questionnaires over 1 year in early middle-aged adults: the CARDIA study. *Sleep*.

[27] Johnson M. (2007). Transcutaneous electrical nerve stimulation: mechanisms, clinical application and evidence. *Reviews in Pain*.

[28] Humphries S. A., Johnson M. H., Long N. R. (1996). An investigation of the gate control theory of pain using the experimental pain stimulus of potassium iontophoresis. *Perception & Psychophysics*.

[29] Maayah M., Al-Jarrah M. (2010). Evaluation of transcutaneous electrical nerve stimulation as a treatment of neck pain due to musculoskeletal disorders. *Journal of Clinical Medicine and Research*.

[30] Martimbianco A. L. C., Porfirio G. J., Pacheco R. L., Torloni M. R., Riera R. (2019). Transcutaneous electrical nerve stimulation (TENS) for chronic neck pain. *Cochrane Database of Systematic Reviews*.

[31] Sun Z. R., Yue J. H., Tian H. Z., Zhang Q. (2014). Acupuncture at Houxi (SI 3) acupoint for acute neck pain caused by stiff neck: study protocol for a pilot randomised controlled trial. *BMJ Open*.

[32] Di Z., Jiang S., Lin X. M., Fu W. B. (2014). The short-term and long-term effects on neck pain caused by cervical spondylosis treated with combination of acupuncture and moxibustion with seed-sized moxa cone. *Zhongguo Zhen Jiu*.

[33] Morikawa Y., Takamoto K., Nishimaru H. (2017). Compression at myofascial trigger point on chronic neck pain provides pain relief through the prefrontal cortex and autonomic nervous system: a pilot study. *Frontiers in Neuroscience*.

[34] Alghadir A. H., Iqbal A., Anwer S., Iqbal Z. A., Ahmed H. (2020). Efficacy of combination therapies on neck pain and muscle tenderness in male patients with upper trapezius active myofascial trigger points. *BioMed Research International*.

[35] Buyukturan B., Guclu-Gunduz A., Buyukturan O., Dadali Y., Bilgin S., Kurt E. (2017). Cervical stability training with and without core stability training for patients with cervical disc herniation: a randomized, single-blind study. *European Journal of Pain*.

[36] Ylinen J., Takala E. P., Nykanen M. (2003). Active neck muscle training in the treatment of chronic neck pain in women: a randomized controlled trial. *JAMA*.

[37] Gattie E., Cleland J. A., Pandya J., Snodgrass S. (2021). Dry needling adds No benefit to the treatment of neck pain: a sham-controlled randomized clinical trial with 1-year follow-up. *Journal of Orthopaedic & Sports Physical Therapy*.

[38] Fernandez-De-Las-Penas C., Plaza-Manzano G., Sanchez-Infante J. (2021). Is dry needling effective when combined with other therapies for myofascial trigger points associated with neck pain symptoms? A systematic review and meta-analysis. *Pain Research and Management*.

[39] McMurtry C. M., Taddio A., Noel M. (2016). Exposure-based Interventions for the management of individuals with high levels of needle fear across the lifespan: a clinical practice guideline and call for further research. *Cognitive Behaviour Therapy*.

[40] Zheng H., Qin B., Yang F. (2015). Lidocaine injection in the intramuscular innervation zone can effectively treat chronic neck pain caused by MTrPs in the trapezius muscle. *Pain Physician*.

[41] Hermanns H., Hollmann M. W., Stevens M. F. (2019). Molecular mechanisms of action of systemic lidocaine in acute and chronic pain: a narrative review. *British Journal of Anaesthesia*.

[42] Lipinski B. (1977). Biological significance of piezoelectricity in relation to acupuncture, Hatha Yoga, osteopathic medicine and action of air ions. *Medical Hypotheses*.

[43] Zhao Z. Q. (2008). Neural mechanism underlying acupuncture analgesia. *Progress in Neurobiology*.

[44] Wei T. H., Hsieh C. L. (2020). Effect of acupuncture on the p38 signaling pathway in several nervous system diseases: a systematic review. *International Journal of Molecular Sciences*.

[45] Chang S., Ryu Y., Fan Y. (2019). Involvement of the cuneate nucleus in the acupuncture inhibition of drug-seeking behaviors. *Frontiers in Neuroscience*.

[46] Fu Z., Hsieh Y. L., Hong C. Z., Kao M. J., Lin J. G., Chou L. W. (2012). Remote subcutaneous needling to suppress the irritability of myofascial trigger spots: an experimental study in rabbits. *Evidence-Based Complementary and Alternative Medicine*.

[47] Langevin H. M., Churchill D. L., Cipolla M. J. (2001). Mechanical signaling through connective tissue: a mechanism for the therapeutic effect of acupuncture. *The FASEB Journal*.

[48] Hsieh Y. L., Chou L. W., Joe Y. S., Hong C. Z. (2011). Spinal cord mechanism involving the remote effects of dry needling on the irritability of myofascial trigger spots in rabbit skeletal muscle. *Archives of Physical Medicine and Rehabilitation*.

[49] Jahre H., Grotle M., Smedbraten K., Dunn K. M., Oiestad B. E. (2020). Risk factors for non-specific neck pain in young adults. A systematic review. *BMC Musculoskeletal Disorders*.

