# Supplementary material for: Rapid Improvement in Neck Disability, Mobility, and Sleep Quality with Chronic Neck Pain Treated by Fu's Subcutaneous Needling: A Randomized Control Study
Source: Pain Res Manag. 2022 Sep 30;2022:7592873. doi: 10.1155/2022/7592873 (PMC9553660; doi:10.1155/2022/7592873)
Supplement: Supplementary Materials — Supplementary Table 1. Difference comparison of NDI in two groups. Supplementary Table 2. Difference comparison of PSQI in two groups. [file 7592873.f1.docx]

**Supplemental Material for the Original Article entitled**

**Rapid Improvement in Neck Disability, Mobility and Sleep Quality with Chronic Neck Pain Treated by Fu`s Subcutaneous Needling: A Randomized Control Study**

**Supplementary Table 1. Difference comparison of NDI in two groups.**

|  | FSN | TENS | *P* value |
| --- | --- | --- | --- |
| Day 8 | -1.60 ± 3.19 | -1.83 ± 4.57 | 0.819 |
| Day 15 | -3.46 ± 4.32 | -2.80 ± 4.35 | 0.554 |

**Supplementary Table 2. Difference comparison of PSQI in two groups.**

|  | FSN | TENS | *P* value |
| --- | --- | --- | --- |
| Day 8 | -0.23 ± 1.88 | -0.53 ± 2.71 | 0.621 |
| Day 15 | -0.73 ± 1.76 | -0.53 ± 2.50 | 0.722 |
